# The Connecticut Valley and Massachusetts Dental Society

**Published:** 1888-12

**Authors:** I. G. Baumgardner


### THE CONNECTICUT VALLEY AND MASSACHUSETTS DENTAL SOCIETY.—UNION MEETING, JULY, 1888.

REPORTED FOR THE "INDEPENDENT PRACTITIONER," BY I. G. BAUMGARDNER, D.D.S.—(CONCLUDED).

Paper read by Dr. B. A. R. Ottolingui, of New York city, on "The Esoteric Law of Cure."

Dr. Ottolingui, by way of an introductory to his paper, said:

"The only apology I have for presenting this paper is that dentists are sometimes willing to hear of things outside the pale of dentistry. The question was raised whether dentists were allopathists or homœopathists, and in this paper I desire to maintain that we belong to no medical school or system, and therefore have the right to use anything that is best." *

The only remark on the paper by way of discussion was by

*Dr. Geo. F. Eames*, Boston, Mass., who said: "There is no such thing as allopathy. It is the old school of medicine, and the platform is as broad as any can be; also the system of giving medicines, including physiological actions of medicines and the fixation of disease by experimental therapeutics to a certain degree. I object to the term 'allopathy' as designating the old school of medicine. All regular physicians object to it."

A committee appointed to investigate Dr. McLean's method of sharpening instruments report that Dr. McLean claimed that he could give a greater cutting power by his process of sharpening than by any other known; and after an examination of the instruments and tools shown them by Dr. McLean, they endorsed the claim.

Dr. McLean was then formally invited to present his method to the society.

*Dr. McLean*—The method which I shall show you to-day may seem very simple to most of you. It, however, did not come to me at once; but is the result of a long series of experiments, and is one which I have now practiced for a number of years. My method is simply to use emery paper of different degrees of fine

---

Dr. Ottolingui accomplished his task fully to the satisfaction of the audience; but as the paper was not of a character suited to our columns, we have not included it in the report.—Ed.

ness, running from 0–00–000–0000, according to the fineness of the edge I wish to produce. For small instruments, I cut out disks from emery paper, same size as the sand-paper disks in general use for polishing fillings. I first place on the mandrel in the engine a disk cut from some thin metal of same size as the emery paper disk; and on this metal disk a similar sized disk cut from thin pasteboard, and on this is placed the emery paper disk. The secret of the process is always to hold the instrument at the same angle against the disk, and to run the disk away from the cutting edge; and, as soon as the feather edge appears, to keep using a finer grade of emery paper until it disappears. Then you will have a perfect edge. The reason why this gives a better edge than a stone is that the stone wears away under the instrument by long use, thus forming grooves or irregularities; so that it is impossible always to have an even surface under the instrument. The emery paper being flexible, and the pasteboard under it acting as a cushion, always keeps an even surface under the instrument.

Another important advantage to be obtained by this method is that the edge of the instrument is always in view, and by watching carefully the formation of a "feather edge" may be avoided. This cannot be done on a stone where water, oil or lather is used. The consequence is that we go past the point where the right degree of sharpness is obtained and the "feather edge" is produced.

I have tried emery paper made by different manufacturers, but have not found any that cuts as well as that made by "Hubert," which is a French paper.

For sharpening large tools I have a machine made like a lapidary's wheel, that runs a large disk which runs horizontally. By having the disk so run you can keep a better watch of the edge of the instrument, as it is always in plain view.

For sharpening burs I use hard rubber corundum disks, grinding the edge of the disk thin by running it against the emery paper. The corundum disk is placed in the engine and the bur held in the hand. I find a jeweler's magnifying glass aids materially in judging when the edges are perfect. Run the disk through the grooves in the bur. A very important point is not to let the burs and instruments get very dull before sharpening. By sharpening every day there will be no trouble about keeping your instruments in good order and will take but little time. You can easily run over all your instruments in quarter of an hour. [Dr. McLean then gave a practical demonstration of his method, sharpening both burs, excavators, ordinary vulcanite scrapers and pocket knives, until they were sharp enough to split hairs or shave with.]

*Dr. Emerson.*—I wish to endorse the claims made by Dr. McLean. I have used instruments sharpened by him for sometime. I did not know his process before, but I *knew* that the instruments sharpened by him cut better than any others I had ever used.

*Dr. Maxfield.*—A paper which I read at Montreal last summer gave a very simple process for making these hard rubber corundum disks alluded to by Dr. McLean. This paper was published in the INDEPENDENT PRACTITIONER for October, 1887, and has since appeared in three or four other journals published in this country and in the *British Journal of Dental Science*, published in London, copied from the former journal. The expense of making these disks is very slight.

*Dr. S. G. Stevens*—I would like to present for your examination a new head-rest which I have recently had made. It is made of wire, woven together similar to the woven-wire mattresses. I find it the most comfortable of anything ever used for a head-rest. Arrangements are being made to place it on the market. I will pass it around for your inspection.

*Dr. Andres*—I would like to call attention to a process for tempering instruments. Every dentist should know how to temper his own instruments; and of the many methods of doing this I think this one the best. Take the crystals of cyanide of potassum, melt them in an iron crucible. Heat the instruments in this liquid and then dip them into a solution of silver, such as is used for silver plating. By doing this you will get an instrument that will stand better than if tempered by any other method.

*Dr. Waters*—I would like to utter a word of caution in regard to using this process. The cyanide of potassum is a violent poison, and great care must be used to prevent inhaling the fumes. I do not think it is a safe process to use.

*Dr. Stanton*—The same result can be accomplished by using the ordinary potash. Heat the instruments to a red heat and plunge into crystals of potash. Heat to a bright cherry for common cutting instruments, and there is no need drawing of the temper afterwards.

*Dr. Searle*—There is nothing new about the process. It was brought before the Connecticut Valley Dental Society and discussed by them over twenty years ago.

*Dr. Waters*—When I was a boy in my teens, the making of penknife blades for my friends was a great pleasure, and the blades I used to make were considered better than those they could buy. My method of tempering was this, the blade was first prepared and brought to an edge. I then would warm it and stick it into a piece of soap, then I would heat it to a cherry red and plunge it into water. The surface would come out perfectly clean. I would then draw the temper to the degree desired.

Dr. J. E. Stanton presented a simple arrangement for making a gas furnace for continuous gum work. The entire expense of which did not exceed three dollars. A full description will be published later.

The committee appointed to investigate the merits of Dr. Harwood's nitrous oxide blow pipe, which he also gave to the profeswithout any restrictions, reported that for utility, simplicity of construction, and economy in use, they felt no hesitancy in fully endorsing it.

*Dr. R. R. Andrews*—Mr. President, in order that we may show some appreciation for what has been given to the profession at this meeting, I would like to introduce the following resolution:

Resolved, That a resolution of thanks engrossed on parchment and signed by the officers of the two societies be presented to Dr. J. E. Stanton, Dr. E. P. McLean and Dr. G. G. Harwood, for their liberal donation of these inventions to the profession.

Unanimously adopted.

The society then listened to Dr. Merriam's closing address, which was as follows:

" The time has now come for the meeting to adjourn, as it was Dr. Andrew's share to bid you welcome, it is now mine to speak the good, old word good-bye.

" The French nobleman's answer when told that 'much would depend on Providence' comes to my mind now. 'Providence,' said he, 'should think well before it sets itself against a gentleman of my position.' Providence has certainly thought all the time of this meeting, for the only interruption of the fine weather that we have had has been in the night time, when poor tired men could rest.

"The reception and kindly feeling that Boston has for all I need not call to mind. But it is with great pleasure that she receives here the society of remarkable geographical boundaries, The Connecticut Valley Dental Society. We feel that it must change its name and be known or described as The Alaska and St. Augustine Dental Society.

"We shall watch its future growth with great interest, and shall not be surprised to hear that at some future meeting that they are to excavate and fill the Mammoth Cave and have yoked Niagara to their dental engine, and have the National Bridge of Virginia among the specimens presented.

"But their presence with us touches a deeper chord. For they bring with them guests from over the Canada border, their friends and ours; being united with them in membership, with us now in friendship.

"Gentlemen who come to us not, as many Americans go over the border, and who are best described as Cowper did the convicts in Australia.

> "'True patriots, for be it understood,
> They left their country for their country's good.'

These friends come for our good and very great pleasure.

"We can but give them this parting word: May the Society and their guests continue to work together; may the Society increase until it is given the heathen for an inheritance, and the uttermost parts of the earth for a possession.

> "'So may the girdle of the sun,
> Bind the east and west in one.'
>
> "'Till Mount Shasta's breezes fan
> The snowy peaks of Ta Siene-Shan,—
> Till Erie blends its waters blue
> With the waves of Tung-Ting Hu,—
> Till deep Missouri lends its flow
> To swell the rushing Hoang Ho!'

"Our meeting has not been one of pleasure merely. I trust I speak for you when I say that there have been some features that will produce a lasting impression on us all.

"You have expressed by your vote the thanks of the meeting for the generous gift to us of the free use of this building, and have voted your thanks to the committees who have served us all so well. But I cannot pass over the fact that this has been in one way a most remarkable meeting.

"Three gentlemen, Dr. J. E. Stanton, Dr. E. P. McLean, and Dr. Geo. F. Harwood, have from pride in their own manhood, and from the love of the profession that was in their hearts, come forward and presented to the profession freely the results of their investigations—results which sold to patent companies, or held as trade-secrets, might have helped to hold the profession in ignorance or unprofessional relations with dealers.

"I cannot believe that this will soon be forgotten; for I am sure that each will be proud of the reception you gave them. All of us are proud to have given it, and look with great pride on them, and on our profession that produced them.

"This cannot, must not be forgotten.

"The Athenian races were not less sharply contested that the prize was only the laurel wreath, and when the profession, either in a meeting like this, or better in a national academy formed for the purpose, places on the brow of those who serve her with honor, by giving to humanity and the world that which increases our knowledge and power, the recognition of faithful service. We say to dental college deans and professors, that there are nobler occupations than 'depot steering,' other avenues to fame than the advertising pages of trade journals, and higher rewards than the dishonoring dollar of the patent dealer.

"I said just now that you had thanked all committees; pardon me if I bring before you again the labor done by one; that of the Committee on Exhibits. I know the work done by each; but it has been my own to aid that one, so that I know of the faithful service of Dr. S. G. Stephens and Dr. W. E. Page, and feel that I may congratulate them again for you.

"It was my pleasure to turn over to Dr. Page what I gathered, and he has systematized and arranged it. I feel, too, that the exhibition spirit should be recognized. For, when a year or more ago, members of the profession visiting New York learned that exhibition privileges had been refused to some, without organization or plan, they each determined on their return, to right matters, that is as far as Boston was concerned; and as they met, each aided the other to open the doors to all who wished to serve the profession, and may they never be closed so long as we have exhibitions.

" This standing rule regarding them has been adopted by both societies to prevent their being degraded to unprofessional or exclusive purposes.

" No committee or officer of the society shall grant to any exhibitor exclusive privileges of exhibition or dispose of exhibit space to one firm, dealer or union of dealers in such way as to exclude or prevent free competition, or in any way hinder *any* having for sale articles of use to the profession from receiving *equal opportunities* of *exhibition*."

" You will notice that this secures to the largest exhibitor equal rights without denying to the maker of one article equality with him. And makes a society unprofessional who would dispose of all space to one individual or firm, because it gives to them power to exclude all others if they wish, and a society cannot escape blame for unprofessional results that it allows to pass unrebuked. It provides that the exhibitions shall be for the profession; for those who do not come to sell their article, but who wish to lease only, cannot claim to be dealers, or that they desire to serve any but themselves.

" The profession is to be congratulated, also that a movement has been begun against 'the combination.' The control of our requirements, those things which our needs create, cannot be allowed to pass from us. We control and need have no fear of the future, if we provide that all who wish to serve us are recognized, and are given opportunity. Our professional honor is stained if we do not protect our individual workman or lend ourselves or our societies to build a prohibitory wall against one poor man or man of small means.

" I have always wanted to get my friend President Andrews into just this position, where we can acknowledge that the microscopist is at the top of the professional ladder, and call his attention to the fact that we are looking well to the foundation on which it rests; for it is manifest that unless the profession controls the requirements of its practice, the microscopist worketh but in vain.

" This then is a sketch of what we have done. As we part today to go back to life's ceaseless toil and endeavor, let us carry with us to refresh and cheer us memories of the days passed together in Boston.

" ' And though fate may throw between us,
The mountains or the sea,
No time shall ever wean us
Nor distance set us free.' "

